# Identification of Underlying Inflammation in Vogt–Koyanagi–Harada Disease with Sunset Glow Fundus by Multiple Analyses

**DOI:** 10.1155/2019/3853794

**Published:** 2019-10-03

**Authors:** Toshihiko Murata, Nanae Sako, Kei Takayama, Kozo Harimoto, Koji Kanda, Carl P. Herbort, Masaru Takeuchi

**Affiliations:** ^1^Department of Ophthalmology, National Defense Medical College, Tokorozawa, Japan; ^2^Centre for Ophthalmic Specialised Care, Lausanne, Switzerland; ^3^University of Lausanne, Lausanne, Switzerland

## Abstract

**Purpose:**

To evaluate underlying subclinical ocular inflammation in Vogt–Koyanagi–Harada (VKH) disease with sunset glow fundus (SGF) by multiple analyses.

**Study Design:**

Retrospective observational study.

**Methods:**

Clinical records of 34 eyes of 17 VKH patients with SGF in whom laser flare photometry (LFP), enhanced depth imaging optical coherence tomography (EDI-OCT), and indocyanine green angiography (ICGA) were performed on the same day were reviewed. The mean age was 57.3 ± 16.3 years, and the mean duration from the initial onset of uveitis was 47.1 ± 22.1 months. Flare counts, ICGA scores, and subfoveal choroidal thickness (SFCT) were compared between eyes.

**Results:**

Although clinical ocular inflammation was observed only in 4 eyes (11.8%), inflammatory signs were observed in 23 out of 34 eyes by LFP (67.6%), in 27 eyes by ICGA (79.4%), and in 10 eyes by SFCT (29.4%). Active inflammatory signs detected by ICGA were observed in 77.8% by LFP and in 25.9% by SFCT. The strength of agreement (Cohen's kappa coefficient) between positive ICGA score and positive flare score was 0.406 (95% CI: 0.076–0.7359, *P* < 0.01), but there was no association between positive ICGA score and increased SFCT. In addition, positive flare count was the significant prognostic factor of positive ICGA score with odds ratio 11.7.

**Conclusions:**

Subclinical ocular inflammation signs were detected in most VKH patients with SGF by ICGA and a substantial proportion of which were also detected by LFP, whereas SFCT was less sensitive to detect subclinical inflammation.

## 1. Introduction

Vogt–Koyanagi–Harada (VKH) disease is an inflammatory disease characterized by a bilateral, diffuse granulomatous panuveitis taking its origin from stromal choroiditis, accompanied by meningeal or neurologic manifestations consisting of headache and meningismus, and auditory disturbances including tinnitus, hearing loss, and vertigo [1, 2]. Ocular manifestations include iridocyclitis, serous retinal detachment, diffuse choroidal thickening, and hyperemia of the optic disk at initial onset [1]. The pathogenesis is considered to be a cell-mediated, autoimmune reaction against melanin-associated proteins in melanocytes in tissues [3]. The prevalence of the disease varies among different countries and ethnics, and pigmented races are more affected than nonpigmented races. In Japan, VKH disease is one of the common causes of uveitis, and it has been reported to be responsible for 7.0% of uveitis patients [4].

Pulse or high-dose steroid therapy followed by slow tapering is the standard regimen for initial onset VKH disease [1, 5, 6]. However, in some VKH patients, even if aggressive use of systemic corticosteroids is properly provided, progression into chronic recurrent granulomatous uveitis occurs with depigmentation of the fundus called “sunset glow fundus (SGF)” [6–8].

Imaging modalities, such as indocyanine green angiography (ICGA) and enhanced depth imaging optical coherence tomography (EDI-OCT), provide critical information to depict choroiditis in VKH disease [9]. ICGA signs in VKH patients include early hyperfluorescent choroidal vessels, fuzzy indistinct large choroidal vessels (choroidal vasculitis), disc hyperfluorescence in severe disease, and hypofluorescent dark dots (HDDs) indicating stromal foci (granulomas) [10], that enable to detect early subclinical disease as well as subclinical recurrences during tapering of therapy [11]. EDI-OCT is a noninvasive modality that allows to investigate the choroidal structures and gives quasi-quantitative measurements of the choroidal thickness. Increase of subfoveal choroidal thickness (SFCT) by EDI-OCT has already been described in the early and chronic recurrent stages of VKH and is an indicative sign of disease activity [12–14]. On the other hand, anterior inflammation is present in VKH disease at late initial disease and during recurrences [2, 15]. On the other hand, aqueous flare is an inflammatory parameter of anterior chamber inflammation resulting from disruption of the blood-ocular barriers. Laser flare photometry (LFP) is a useful device to quantify aqueous flare associated with intraocular inflammation objectively with high reproducibility [16, 17]. Furthermore, LFP allows detection of subclinical disruption of the blood-ocular barriers due to subtle pathological alternations.

Although ocular inflammation subclinically progressing in chronic VKH disease with SGF is sight-threatening, the detection is still challenging. However, it is crucial for the management of chronic recurrent VKH disease to detect subclinical choroidal inflammation by multiple analyses in order to achieve global evaluation of the inflammatory involvement of all intraocular structures. In the present study, we compared ICGA findings, SFCT measured by EDI-OCT, and aqueous flare counts by LFP in VKH patients with SGF and evaluated underlying inflammation and the correlation between these analyses.

## 2. Subjects and Methods

This is a retrospective uncontrolled cross-sectional study. Clinical records of 34 eyes of 17 VKH patients with SGF in whom LFP, EDI-OCT, and ICGA were performed in the same day at the National Defense Medical College Hospital from January 1 to July 31, 2018, were reviewed. The National Defense Medical College Hospital Ethics Review Board (ERB) approved this retrospective analysis of patient data (2767). The study protocol was described to all human subjects, and written informed consent was waived by the ERB due to the retrospective nature of the study. The research was conducted in accordance with the tenets set forth in the Declaration of Helsinki. Following the revised criteria of VKH disease [18], all the patients were diagnosed as incomplete VKH disease. Exclusion criteria were presence of corneal diseases, primary glaucoma, exfoliation syndrome, history of trauma or vitrectomy, history of other uveitis, other systemic inflammatory disease, or malignancy. Age, sex, best-corrected visual acuity, duration of disease and clinical findings of disease activity at the time of examination, flare counts measured by LFP, EDI-OCT measurements, ICGA signs, systemically administrated total corticosteroid amounts, additional treatment with cyclosporine, and follow-up details were recorded. Best-corrected visual acuity (BCVA) was expressed as the logarithm of mean angle of resolution (logMAR).

### 2.1. Aqueous Flare Counts

Aqueous flare was measured using the Kowa FM 700 LFP (Kowa Company Ltd., Nagoya, Japan). Eight flare counts were taken for each eye and averaged after excluding the minimum and maximum measurements in each series of counts. A single averaged count for each eye was produced. Since flare counts of normal eyes are from 4 to 6 ph/ms [19], the cutoff value was determined at 10 ph/ms to include only patients with significant subclinical inflammation.

### 2.2. Measurement of Subfoveal Choroidal Thickness

The EDI-OCT images were acquired using the Spectralis HRA + OCT (Heidelberg Engineering Inc., Heidelberg, Germany). The posterior segment centered on the fovea was scanned by 8.8 mm × 7.3 mm at 240 *μ* intervals at 8.8 images per second, with a resolution of 5 *μ*m. SFCT on EDI-OCT images was defined as the vertical distance between the outer surface of the RPE and the choroidal-scleral interface (CSI) at the fovea. The cutoff value was set by 280 *μ*m which is the average of SFCT of normal eyes [20].

### 2.3. ICGA Images

ICGA was performed after an intravenous injection of 25 mg of indocyanine green (Santen Pharmaceutical Co., Ltd., Osaka, Japan). Ultra-widefield ICGA images were captured using an ultra-wide-field imaging device (Optos California ultra-wide-field imaging device; Dunfermline, Scotland, UK). Sequential time-stamped images were obtained for each eye during transit and completion of choroidal filling. Images were digitally captured using the Optos V2 Vantage Review Software and subsequently compressed into high-quality JPEG/TIFF files. Using the dual FA/ICGA scoring system established by the Angiography Scoring for Uveitis Working Group [21], ICGA signs were scored in a masked fashion by 3 ophthalmologists, and the averages were used. Four was set up for the cutoff value.

### 2.4. Statistical Analysis

Chi-squared and Mann–Whitney *U* tests were used to compare the results between the two groups. Correlations between two numerical factors were analysed by Spearman's rank correlation coefficient, and those of two categorical factors were analysed by Cohen's kappa coefficient. Prognostic factors of positive ICGA score were examined by logistic regression analysis. *P* values > 0.05 were considered statistically significant.

## 3. Results

### 3.1. Main Characteristics and Clinical Features

Table 1 shows characteristics and clinical features of VKH patients with SGF. Of 17 VKH patients with SGF enrolled, the age (mean ± standard deviation) at examination was 57.3 ± 16.3 years (range 29–81 years), and 8 (47.1%) were men and 9 (52.9%) were women (Table 1). The mean duration from the initial onset of uveitis was 47.1 ± 22.1 months (range 20–85 months), and the mean logMAR was −0.15 ± 0.09 months (range −0.18 to 0.4). The mean amount of systemic corticosteroids administrated from the initial onset of VKH disease was 8190 ± 5956 mg (range 0–18350 mg) by prednisolone conversion. Three patients had not been treated with systemic corticosteroids, of which 2 patients (CVKH 2 and 10) had only mild ocular inflammation and 1 patient (CVKH9) had diabetes and osteoporosis. Cyclosporine and subtenon injection of triamcinolone acetonide were used for treatment of CVKH9 instead of systemic corticosteroids.

### 3.2. Flare Counts, ICGA Scores, SFCT, and Clinical Ocular Inflammation in Individual VKH Patients with SGF

Inflammatory parameters of individual VKH patients with SGF are shown in Table 2. Clinical ocular inflammation was observed in 4 out of 34 eyes (CVKH 10, 11, and 16), in which anterior ocular inflammation was observed in all these eyes and one eye presented SRD. However, inflammatory signs were observed in 23 out of 34 eyes by LFP (67.6%), in 27 eyes by ICGA (79.4%), and in 10 eyes by SFCT showing increased thickness (29.4%). In 4 eyes with clinical inflammation, flare counts were positive in all four and an increased ICGA score was present in 3 eyes, but increased SFCT was observed only in one eye. In addition, SFCT was not increased in the eye with SRF.

### 3.3. Correlations between Inflammation Parameters

Table 3 shows sensitivity, specificity, positive predictive value, and negative predictive value of positive flare count and increased SFCT for positive ICGA score. Sensitivity, specificity, positive predictive value, and negative predictive value of positive flare count were 77.8%, 71.4%, 91.3%, and 45.4%, and those of increased SFCT were 25.9%, 57.1%, 70.0%, and 16.7%, respectively. All values of flare count were higher than those of SFCT. The strength of agreement (Cohen's kappa coefficient) between positive ICGA score and positive flare score was 0.406 (95% CI: 0.076–0.7359, *P* < 0.01), but that between positive ICGA score and increased SFCT was −0.089 (95% CI: −0.309–0.1308, *P*=0.191). Subsequently, logistic regression analysis was conducted in which the dependent factor was positive ICGA score and independent factors were positive flare count and increased SFCT. As shown in Table 4, positive flare count (*P*=0.0315; odds ratio 11.7; 95% confidence interval: 1.243–109.8) was identified as the prognostic factor of positive ICGA score.

### 3.4. Correlations between Duration from the Initial Onset of Uveitis and Inflammatory Parameters

**Figure 1:**
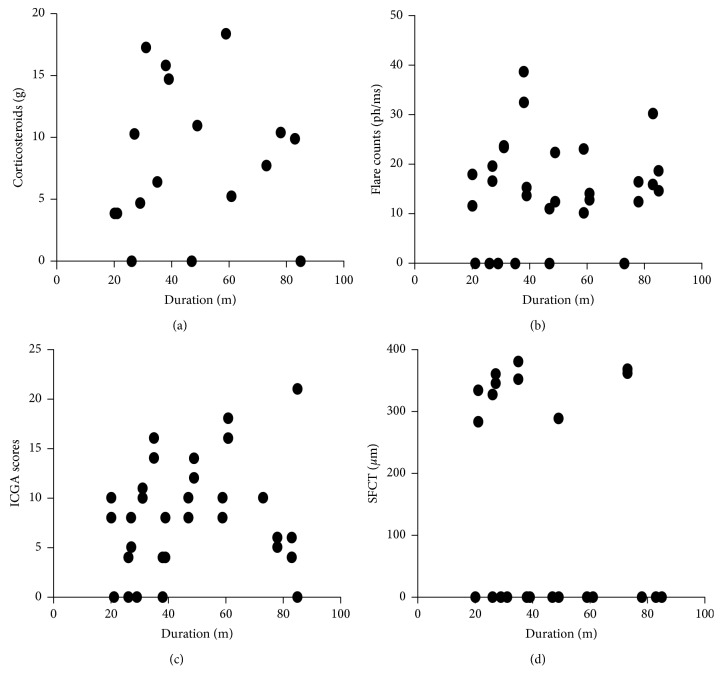
Association of duration from the initial onset of uveitis with clinical features. Correlation of duration from the initial onset of uveitis (months) with (a) total amounts of systemic corticosteroids (*y*=7.75+9.37*x*, *R*_2_=0.0012, *P*=0.845), (b) flare count (*y*=11.5+0.057*x*, *R*_2_=0.01994, *P*=0.4257), (c) ICGA score (*y*=5.08+0.059*x*, *R*_2_=0.058, *P*=0.1689), and (d) SFCT (*y*=281 − 0.78*x*, *R*_2_=0.0472, *P*=0.2171) in VKH patients with SGF was analysed by Spearman's rank correlation coefficient. “0” represents flare < 10; SCT < 280, and ICGA score < 4.

Figure 1 shows correlation of duration from the initial onset of uveitis with total amounts of systemic corticosteroids, flare count, ICGA score, and SFCT. There was no statistically significant correlation between duration from the initial onset of uveitis and any of these factors.

## 4. Discussion

Detection of choroidal inflammation subclinically progressing in VKH disease with SGF is still challenging. In this study, ocular inflammatory signs associated with chronic VKH disease were depicted in 79.4% of the eyes by ICGA, in 67.6% of the eyes by LFP, and in 19.4% of the eyes by SFCT although further clinical investigation is required to determine whether treatment should be performed for these subclinical inflammations.

There are different evolutional stages in VKH disease from the initial onset to the chronic stage which possess varying clinical presentation, course, and prognosis. At the onset of VKH disease, the first prodromal stage of disease is usually lasting for a few days/weeks that corresponds to subclinical inflammation initiating exclusively in the choroidal stroma at the ocular level. The initial choroidal involvement without other ocular signs can only be detected by ICGA [11], or possibly by EDI-OCT. Subsequently, the disease becomes clinically apparent when choroidal inflammation spreads into the surrounding tissues including the optic disc, the retina, the ciliary body, and the anterior chamber. In this second stage, classical clinical signs are apparently presented by bilateral papillitis, bilateral serous detachments of the retina, and mild to moderate anterior inflammation seen like a nongranulomatous uveitis [7, 22], which are able to be identified by LFP, FA, and EDI-OCT in addition to ICGA. If the proper treatment with aggressive use of systemic corticosteroids and nonsteroidal immunosuppression is not provided at this early stage of the disease onset, the intraocular inflammation proceeds to the chronic stage characterized by recurrent granulomatous uveitis with typical SGF [22–25]. The chronic disease results from late or insufficient treatment of initial-onset disease and presents a different clinical course, being more vulnerable to recurrences and more resistant to treatment [7, 26, 27].

Our study deals with this chronic situation. Keino et al. have reported that among 80 VKH patients treated with high-dose corticosteroid therapy from the initial onset, 67.5% of patients presented SGF although apparent chronic clinical ocular inflammation occurred only in 17.5% of the patients [28]. As well, Sakata et al. indicated that approximately 80% of VKH patients, who were treated with early high-dose corticosteroids within 1 month from disease onset followed by slow taper (at least 6 months), progressed to chronic recurrent disease [8]. Other studies also showed that only high-dose corticosteroids at the disease onset is not sufficient, being unable to prevent evolution of chronic or recurrent granulomatous inflammation with SGF, peripapillary atrophy, and depigmented small atrophic lesions at the level of retinal pigment epithelium [7, 29]. In VKH patients with SGF of this study, although most of the patients were treated with high-dose corticosteroids at the disease onset, subclinical chronic recurrent inflammatory features were detected in 79.4% of the eyes by ICGA, while only 11.8% of the eyes presented clinical ocular inflammation.

SFCT is a useful parameter to evaluate choroidal inflammation in the acute phase of VKH disease. However, Jap and Chee reported that SFCT positively correlated with ICGA score in the initial onset of VKH patients, but negatively correlated with age and duration of disease of VKH patients with 6 or more months after the disease onset [30]. Therefore, its utility in detecting any potential alternations of chronic recurrent VKH disease is limited [9, 13]. The present study indicated that sensitivity of SFCT against active inflammatory signs in chronic VKH patients with SGF detected by ICGA was only 25.9%, and increased SFCT was not the significant prognostic factor of positive ICGA score. EDI-OCT allows us to investigate choroidal structures of the posterior pole including quasi-quantitative measurements of the choroidal thickness; however, stromal choroiditis during a process of VKH disease involves the entire fundus [9]. In addition, since the magnitude of the change in SFCT during recurrence of chronic VKH with SGF is much lower than that occurring during the acute disease, it is considered that the slight choroiditis could not be depicted by SFCT. It has been reported that significant difference in SFCT changes could not be found between eyes with and without clinical inflammation in long-standing VKH disease patients [13].

Elevation of aqueous flare results from destruction of blood-ocular barriers by the anterior inflammation and by spillover of posterior inflammation. Chronic recurrent VKH disease presents significantly more severe anterior inflammation compared with initial-onset acute VKH disease [7], and flare count is significantly higher in patients with chronic recurrent VKH disease than that in initial-onset acute VKH disease [15]. Yang et al. have indicated that the intraocular inflammation in VKH disease progresses from the posterior segment to the anterior segment and from anterior uveitis presented by nongranulomatous inflammation to recurrent or chronic granulomatous uveitis if appropriate treatment is not provided [22]. In addition, Bacsal et al. demonstrated that recurrence of isolated anterior inflammation in VKH disease can occur concomitantly with subclinical choroidal inflammation [29]. Therefore, LFP is considered to be a crucial device for monitoring of chronic recurrent VKH disease. As well in the present study, inflammatory signs were detected in 67.6% of VKH patients with SGF by LFP, and sensitivity of positive flare count against active inflammatory signs detected by ICGA in chronic VKH patients with SGF was 77.8%, which was significantly more than 25.9% of SFCT (Tables 2–4). Furthermore, positive flare count was the significant prognostic factor of positive ICGA score with odds ratio 11.7. On the other hand, as positive predictive value of flare count for positive ICGA score was not 100%, 2 eyes were positive flare counts but the ICGA scores were less than 4 (VKH10 L and VKH19 R). SFCT was also less than 280 *μ*m in these two eyes (Table 2). Although iridocyclitis was observed in VKH10 L but not in VKH19 R, it is unclear whether those elevated flare counts resulted from irreversible damage of the anterior ocular blood barrier or from ocular inflammation limited in the anterior segment.

In VKH patients with SGF, although visual acuity (VA) is generally preserved, visual function is impaired and abnormal [31]. Abnormal retinal sensitivities of VKH patients with SFG are able to be identified by microperimetry [26]. da Silva et al. reported that choroidal thinning progressed corresponding to disease duration of VKH disease, and SFCT in long-standing VKH disease patients was thinner than that of controls [13]. In the present study, SFCT did not correlate with duration from the initial onset of uveitis (Figure 1). Since SFCT is affected by age, corneal thickness, axial length, IOP, and spherical equivalent [32, 33], it could be due to lack of adjustment for these potential compounding factors in this study.

Limitations of the present study that should be noted are the small number of patients only from Japanese origin, the single center, various onset age and disease duration, and retrospective nature of the study. Further prospective study including more patients from other ethnicities as well as evaluation of disease stages in detail including irreversible ocular features could allow additional comparison of differences in these analyses.

In summary, ICGA revealed active inflammation in most of the VKH patients with SGF and this was also the case to a slightly lesser extent for aqueous flare by LFP. Thickening of SFCT by EDI-OCT was however found in a little more than ¼^th^ and seems to be a less reliable parameter to account for subclinical inflammation. Taken together, these data clearly show that ongoing occult choroidal inflammation is taking place in apparently quiet disease possibly evolving in the long run to decrease of the quality of visual function and complications. The question arises of whether these patients should be followed with the precise measurement methods presently at our disposal and should be treated until resolution of subclinical inflammation is achieved.

## Figures and Tables

**Table 1:** Characteristics and clinical features of VKH patients with SGF.

	Sex	Age	Disease duration (M)	LogMAR (R/L)	IOP	Cor	Cyc
CVKH2	M	51	26	−0.18/−0.18	13/13	0	−
CVKH3	M	33	20	−0.18/−0.08	10/9	3840	+
CVKH4	F	50	59	−0.18/−0.08	12/14	18350	−
CVKH5	F	67	61	0/−0.08	11/15	5245	−
CVKH6	M	68	29	0/0	13/12	4690	−
CVKH7	F	81	49	−0.08/0.05	12/12	10908	−
CVKH9	F	79	47	−0.08/0	17/16	0	+
CVKH10	F	41	85	−0.08/0.08	17/15	0	−
CVKH11	F	54	31	0.22/0.1	11/13	17219	+
CVKH13	M	64	83	−0.18/−0.18	12/13	9873	−
CVKH14	F	37	35	−0.18/−0.08	13/13	6385	−
CVKH15	M	75	73	−0.18/−0.18	14/14	7752	−
CVKH16	M	51	27	−0.18/−0.18	12/11	10255	−
CVKH17	F	53	78	−0.05/−0.18	16/17	10393	−
CVKH18	F	29	21	0.4/−0.18	15/16	3840	−
CVKH19	M	77	38	0.1/0.15	11/11	15805	−
CVKH20	M	64	39	−0.18/−0.18	12/11	14674	−

IOP, intraocular pressure; Cor, corticosteroids; Cyc, cyclosporine.

**Table 2:** LFP, ICGA, and SFCT in individual VKH patients with SGF.

		Flare counts (ph/ms)	ICGA scores	SFCT (*μ*m)	Iridocyclitis	SRD
CVKH2	R	<10	4	<280	−	−
	L	<10	<4	327	−	−

CVKH3	R	11.6	8	<280	−	−
	L	18	10	<280	−	−

CVKH4	R	23.1	8	<280	−	−
	L	10.2	10	<280	−	−

CVKH5	R	14.1	16	<280	−	−
	L	12.8	18	<280	−	−

CVKH6	R	<10	<4	<280	−	−
	L	<10	<4	<280	−	−

CVKH7	R	22.4	14	<280	−	−
	L	12.4	12	288	−	−

CVKH9	R	11.1	8	<280	−	−
	L	<10	10	<280	−	−

CVKH10	R	14.6	21	<280	+	+
	L	18.6	<4	<280	+	−

CVKH11	R	23.3	10	<280	+	−
	L	23.8	11	<280	−	−

CVKH13	R	15.8	6	<280	−	−
	L	30.2	4	<280	−	−

CVKH14	R	<10	14	351	−	−
	L	<10	16	380	−	−

CVKH15	R	<10	10	368	−	−
	L	<10	10	361	−	−

CVKH16	R	19.6	8	360	−	−
	L	16.5	5	345	+	−

CVKH17	R	12.4	5	<280	−	−
	L	16.4	6	<280	−	−

CVKH18	R	<10	<4	334	−	−
	L	<10	<4	283	−	−

CVKH19	R	38.6	<4	<280	−	−
	L	32.5	4	<280	−	−

CVKH20	R	13.7	8	<280	−	−
	L	15.3	4	<280	−	−

ICGA, indocyanine green angiography; SFCT, subfoveal choroidal thickness; AI, clinical anterior inflammation; SRD, serous retinal detachment.

**Table 3:** Correlation of ICGA sign with detection rates of flare count and SFCT measurement.

	Sensitivity (%)	Specificity (%)	Positive predictive value (%)	Negative predictive value (%)
Flare count	21/27 (77.8)	5/7 (71.4)	21/23 (91.3)	5/11 (45.4)
SFCT	7/27 (25.9)	4/7 (57.1)	7/16 (70.0)	1/18 (16.7)

SFCT, subfoveal choroidal thickness.

**Table 4:** Prognostic factors of positive ICGA scores.

	Odds ratio	95% CI	*P* value
Flare count	11.7	1.243–109.8	0.0315
SFCT	1.73	0.185–16.23	0.6297

CI, confidence interval; SFCT, subfoveal choroidal thickness.

## Data Availability

The data used to support the findings of this study are included within the article.
